# Effects of Seed Roasting Temperature on Sesame Oil Fatty Acid Composition, Lignan, Sterol and Tocopherol Contents, Oxidative Stability and Antioxidant Potential for Food Applications

**DOI:** 10.3390/molecules27144508

**Published:** 2022-07-14

**Authors:** Radia Arab, Susana Casal, Teresa Pinho, Rebeca Cruz, Mohamed Lamine Freidja, José Manuel Lorenzo, Christophe Hano, Khodir Madani, Lila Boulekbache-Makhlouf

**Affiliations:** 1 Laboratoire de Biomathématiques, Biophysique, Biochimie, et Scientométrie (L3BS), Faculté des Sciences de la Nature et de la Vie, Université de Bejaia, Bejaia 06000, Algeria; mfreid@hotmail.fr (M.L.F.); madani28dz2002@gmail.com (K.M.); labo3bs@gmail.com (L.B.-M.); 2 Requimte—LAQV, Laboratório de Bromatoologia e Hidrologia, Faculdade de Farmácia, Universidade do Porto, Rua de Jorge Viterbo Ferreira, 228, 4050-313 Porto, Portugal; sucasal@ff.up.pt (S.C.); teresapinho847@gmail.com (T.P.); rcruz@ff.up.pt (R.C.); 3 Département de Biochimie et de Microbiologie, Faculté des Sciences, Université Mohamed BOUDIAF, M’sila 28000, Algeria; 4 Centro Tecnológico de la Carne de Galicia, Rúa Galicia Nº 4, Parque Tecnológico de Galicia, San Cibrao das Viñas, 32900 Ourense, Spain; jmlorenzo@ceteca.net; 5 Área de Tecnología de los Alimentos, Facultad de Ciencias de Ourense, Universidad de Vigo, 32004 Ourense, Spain; 6 Laboratoire de Biologie des Ligneux et des Grandes Cultures, INRAE USC1328, Orleans University, CEDEX 2, 45067 Orléans, France; 7 Bioactifs et Cosmétiques, CNRS GDR3711, CEDEX 2, 45067 Orléans, France; 8 Centre de Recherche en Technologie Agro-Alimentaire, Route de Targua-Ouzemour, Bejaia 06000, Algeria

**Keywords:** sesame oil, roasting temperature, composition, oxidative stability, lipid oxidation, meat products

## Abstract

Roasting is a key step for preparing sesame oil that leads to important changes in its organoleptic properties and quality. In this study, white sesame seeds were roasted for 20 min in an electric oven at different temperatures (120, 150, 180, 210, 250 and 300 °C). The oils extracted from unroasted and roasted seeds were compared for their chemical composition: fatty acids (including trans isomers), phytosterols, lignans (sesamin and sesamolin), tocopherols and total phenolic compounds, as well as their oxidative stability and antiradical capacity. There were no obvious differences in the oil densities, refractive indexes or iodine values, but the saponification values were affected by temperature. Relevant primary and secondary lipid oxidation were observed at T > 250 °C, resulting in a higher *p*-anisidine value and K232 as well as K268 values. Roasting improved oil yield (from 33.5 to 62.6%), increased its induction period (from 5.5 to 10.5 h) and enhanced the total phenolic content (from 152 to 194 mg/100 g) and antiradical activity of the extracted oil. Depending on roasting temperature, a gradual decline was recorded in total amounts of phytosterols (up to 17.4%), γ-tocopherol (up to 10.6%), sesamolin (maximum of 27.5%) and sesamin (maximum of 12.5%). All the investigated oils presented a low quantity in triglyceride polymers, clearly below the maximum tolerated quantity according to the European regulation. The optimal roasting temperature for obtaining high nutritional grade oil within the permissible values was 210 °C. The unsaponifiable components (including lignans and sterols) extracted from roasted seeds have been shown to be natural additives to fresh meatball products to extend shelf life. The results of this study may help to boost the nutritional content of plant-based diets by allowing for the use of roasted sesame seed oil and its components.

## 1. Introduction

Sesame (*Sesamum indicum* L.), also known as gingelly, bennissed, benne, sim-sim and til, belongs to the Tubiflorae order and Pediaceae family, which comprises about 40 species [1,2]. Sesame seeds are a potential source of nutrients, as they contain 35–57% oils, 20–25% proteins, 20–25% carbohydrates and 5–6% minerals [3]. Their oil content is higher than most of the known oil seeds. Sesame oil is regarded as a high-priced and high-quality product. It contains approximately 14% saturated, 39% monounsaturated and 46% polyunsaturated fatty acids [3]. Sesame oil has a long shelf life compared to others vegetables oils, even when stored at ambient temperatures, despite its high degree of unsaturation. This is attributed to endogenous antioxidants which include mainly γ-tocopherol, lignans such as sesamol, sesamolin or sesamin and other phenolic derivatives [4]. Recent studies have reported that the consumption of sesame or sesame products can improve human health, including anti-inflammatory and anticarcinogenic effects, blood pressure and serum lipid-lowering effects, inhibiting in vitro malignant melanoma growth and human colon cancer cell proliferation and increasing the bioavailability of γ-tocopherol [5,6].

Roasting is a key step in the oil industry which results in important physical, chemical, structural and organoleptic changes [7]. This process was reported to increase the yield of extracted oil [8], its lignan contents and its antioxidant activity [9]. The nutritional quality and composition of the oils, as well as their sensory characteristics and acceptability by consumers, are affected by the roasting temperature [10]. During the roasting process, seeds become more crumble and brittle. Changes in roasting temperature have been reported to produce brown pigments. Non-enzymatic browning and phospholipids degradation are responsible for color formation in sesame oil. According to Yoshida et al. [9], increases in roasting temperature caused losses in sesamin (from 7271 to 6902 mg/Kg of oil roasted at 220 °C for 25 min) and sesamolin (from 4605 to 2873 mg/Kg of oil at 220 °C for 25 min). Tocopherol was still present at more than 80% of its original value after roasting for 25 min at all temperatures. The effects of roasting at high temperatures are complex. It causes the liberation of free fatty acids by hydrolysis of mono-, di- and tri-acylglycerols which can accelerate the process of oil oxidation, leading to poor oil quality. It may also result in the production of off-flavors and trans fatty acids, which are both deleterious for human health. In particular, there is growing evidence for a possible role of T-fat in the development of Alzheimer’s disease and cognitive decline with age. [11]. However, roasting also favors the development of colored products, and is reported to possibly participate in the prevention of lipid oxidation [7]. It cleaves and liberates phenolic compounds [12], for instance, the lignan sesamolin can be degraded into other phenolic compounds with higher antioxidant potential [13].

Lipid oxidation is also a chemical reaction that lowers the nutritional value of other food products [14,15]. The quality attributes of meat and meat products are largely affected by lipid oxidation during processing and storage [14,15]. Interestingly, according to a recent report, sesame oil may be a potentially effective natural additive to fresh meat products for prolonging shelf life during cold storage [15]. There are also questions concerning the safety of synthetic antioxidants [14]. As a result, there is an intense search for safe and natural antioxidants that can slow the oxidative deterioration of lipids, improve their quality and keep their nutritional worth.

The current study sought to ascertain: (1) the influence of roasting temperature on sesame seed extracted oil properties, including changes in both composition (fatty acid profile, lignan, sterol tocopherol and triglyceride derivatives) and physicochemical aspects; (2) the oxidative stability and antioxidant capacity, as evaluated in raw and roasted sesame seed oils; (3) the antioxidant potential for food industry applications by evaluating the oxidative stability of meatballs.

## 2. Results and Discussion

### 2.1. Physical and Chemical Parameters

Physical and chemical properties of extracted oil from unroasted (USSO) and roasted (RSSO) seeds at different temperatures are represented in Table 1.

The extraction yield of the oil is related to various factors, such as temperature and moisture. The yield in crude oil before roasting (USSO) was 33.5%. RSSO samples showed significantly higher (*p* < 0.001) oil yields than the USSO one. An increase in roasting temperature resulted in an increase in extraction yield with its maximum (86.6%) at 250 °C. Since, at this temperature, the cell membranes were extensively disrupted, the dry heat improved the mass transfer coefficients of the seeds and facilitated the release of the oil through the increased porosity of the cell wall [16,17]. Roasting sesame seeds at different conditions was previously found to increase oil yields with increasing temperature and time [16]. This might be due to protein denaturation (changes in protein bodies and damage of lipoprotein membranes surrounding lipid bodies) during heating causing damage to the cell membranes, thus improving oil extractability by releasing cellular content through the heat produced by the movement of polar water molecules in the tissue [17]. In our study, roasting may have caused similar changes in the sesame structures.

Because it affects physicochemical and microbiological properties, it is crucial to investigate the moisture level of food products. Because humidity may be the starting point of several chemical reactions and microbial contaminations, it is also an essential parameter for food product preservation.

Based on the results of Table 1, roasting significantly reduces the moisture content in samples compared to the unroasted sample (*p* < 0.001) so that the initial moisture content of seeds decreased from 2.55% to 0.15% in treated samples at temperatures of 300 °C, and among 94% were eliminated under this high temperature. The unroasted sample and treated samples at various temperatures both had low moisture contents, which suggests that these products were stable and resistant to microbial and enzymatic degradation. The elimination of water from seeds will avoid eventual contamination during storage of extracted oils and obtained defatted sesame flour.

The refractive index is mainly used to characterize changes in unsaturation as the oil is hydrogenated; it increases with the increasing level of double bonds [18]. The refractive index for USSO was 1.476, which is near to the value reported for sesame from different origins [17,18,19]. For all of the oils, refractive indexes varied between 1.461 and 1.476, then it decreased with increases in roasting temperature. This is probably due to the polymerization of unsaturated FA in the oils. These changes are statistically significant (*p* < 0.05). The specific gravity for USSO was 0.919, which agrees with that previously found by many authors [18,20]. This physical parameter was not (*p* > 0.05) affected by roasting temperature.

The iodine value (IV) is generally used to measure the degree of unsaturation in fatty acids of triacylglycerols [18,21], solidification temperature and oxidation stability. High values indicate that the oil contains a greater number of double bonds [22]. The iodine value remained quite stable with a value of 113 g I_2_/100 g oil for USSO, and with only a slight decline to 103 g I_2_/100 g in the oil extracted from seeds as a result of roasting at 300 °C. The same result was observed for pumpkin seed oil roasted at different temperatures ranging from 90 to 200 °C [23]. The more unsaturated oils are vulnerable to heat oxidation. The iodine value is frequently employed as a rough indicator of oil stability during roasting. Moreover, the amount of PUFA present also provides additional evidence of stability because they oxidize more quickly than oleic acid, which contains one double bond. Sesame contains more than 44% PUFA (Table 2). As shown in Table 1, the decrease in the iodine value is greater from 250 °C, which may indicate a reduction in the number of double bonds of PUFA and this probability is reinforced by the reduction in the refractive index mentioned above.

The saponification value (SV) is an indicator of average molecular weight (it has an inverse relationship with molecular weight), which gives information on the alkali-reactive groups in oils. The SV of the control sesame oil was 185.4 gKOH/100 g oil, and gradually increased to 200.0 gKOH/100 g when the roasting temperature reached 300 °C. The low saponification value of sesame oil also indicates that it may not be suitable for soap making but is very good nutritionally.

**Figure 1:**
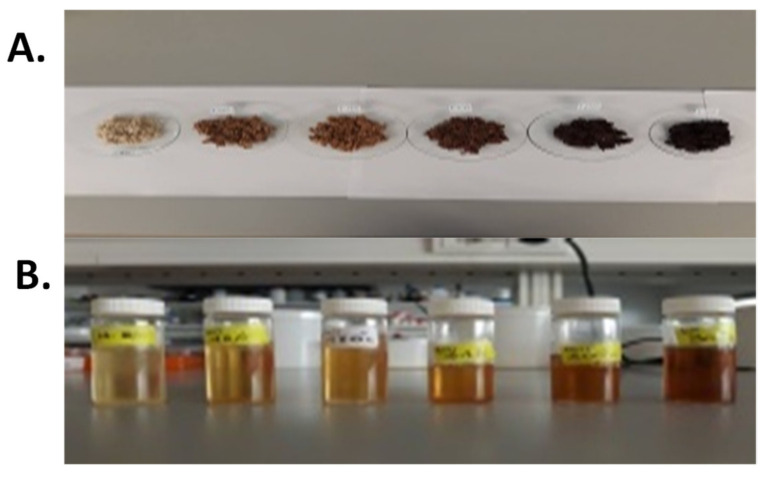
(**A**) Unroasted and roasted sesame seeds at different temperatures (top) and (**B**) their corresponding oils (bottom). From right to left: unroasted, roasted at 150, 180, 210, 250 and 300 °C, respectively.

The visual color of seeds and oils provides information on the intensity of heat treatment. The color development of sesame oils changed gradually from light yellow in USSO to brown (under 210 °C) and finally to deep brown at 300 °C (Figure 1). Globally, seed and oil colors were highly affected (*p* < 0.001) by roasting temperature. The highest lightness (L*) value was recorded in USSO (48.66) and decreased with increasing temperature, leading to a darkening of roasted sesame seed oils. A linear increase in red color (a*) (R^2^ = 0.78, *y* = 0.017 *x* − 4.61) resulting from browning substances such as melanoidin, which may be formed by non-enzymatic Maillard reaction, is observed (Table 1). Similar results were observed with sesame [24].

### 2.2. Analysis of Oil Composition

Fatty acid (FA) composition of USSO (Table 2) was similar to or within previously reported ranges [25,26]. The major FAs recorded both before and after thermal processing were linoleic (ranging from 43.6 to 44.4%), oleic (ranging from 37.0 to 37.6%), palmitic (ranging from 9.2 to 9.4%) and stearic acid (ranging from 6.1 to 6.2%).

USSO and RSSO contained 16.24 to 16.50% saturated fatty acids (SFA), 38.2 to 38.9% monounsaturated and more than 44% polyunsaturated fatty acids (PUFA). Statistically, there was a significant difference (*p* < 0.05) between USSO and RSSO in the profile of FA. This result was in agreement with that previously reported [27].

As it can be seen in Table 2, the relative proportions of SFA (C22:0, C16:0, C18:0) increased while that of the unsaturated one (C18:1, C18:2, C18:3, C20:1) declined. The changes, however, were small and the differences were not statistically significant (*p* > 0.05): a decrease of 0.74% in C18:1; 1.73% in C18:2; and 5.2% in C18:3. Several previous studies had investigated changes in FA and provide useful background. It was shown that the roasting of brown sesame seeds increased SFA proportion, decreased linoleic acid from 46.1% to 24.8% and decreased α-linolenic acid from 0.4 to 0.2% [28]. Contents of oleic and linoleic acids were drastically reduced when roasted at >240 °C for 30 min and the retention in total FA contents of oils prepared by roasting at 240 and 260 °C for 30 min also reduced [29].

Roasting affected (*p* < 0.05) TFAs, and the total content varied from 0.20 to 0.84%. The maximum was recorded in RSSO at 300 °C. Many studies have shown that TFAs can be formed in oil after seed roasting processes. It was observed that contents in TFAs such as C18:2t and C18:3t increased gradually as roasting time increased at all temperatures used with a maximum content of 0.8% in perilla seeds [30]. Production of TFAs including C18:2_t9c12 and C18:2_C9t12 was linear (R^2^ = 0.77; R^2^ = 0.80, respectively), whereas production of C18:2_t9t12 was absent until 250 °C. A similar linear increase (R^2^ = 0.99) was reported as a function of time in sesame seeds [27]. From Table 2 it can be deduced that the isomerization of the double bonds at carbon 9 and 12 begins from 250 °C in linoleic acid. Nevertheless, the presence of TFAs in RSSO is not considered as harmful because their level is under the threshold allowed by the European regulation with a maximum authorized value of 2%.

Quantification of triglyceride (TG) derivatives is one of the most used methods to evaluate the degradation of edible oils, especially those used in frying [31]. As a result, determining the total amount of newly produced TG derivatives in the seeds roasted at high temperatures is important. Results are summarized in Table 3. Total polar compounds expressed in g/100 g of oil ranged from 5.7 to 6.7. The lower value corresponds to USSO while the higher one was exhibited by the RSSO at 300 °C.

As expected, unroasted oil is characterized by a very low (0.1%) level of triglyceride polymers and triglyceride dimmers. Roasting at 150 °C and 300 °C enhanced TGP + TGD by 95.5 and 98.9%, respectively, as well as the contents of oxTGM, DG and FFA (Table 3). Most importantly, all the investigated oils presented a low quantity in triglyceride polymers, clearly below the maximum tolerated quantity according to the European regulation with a maximum authorized value of 14%.

### 2.3. Tocopherols, Phytosterols, Lignans and Other Related Total Phenolic Contents

Only γ-tocopherol was detected among tocopherol homologues in our samples. As has been reported in the literature, 97% of the tocopherol in sesame oil was in the γ-form [3]. USSO contained 25.9 mg/100 g; this quantity was in close agreement with the values previously reported [29,32]. After heat treatment, it was reduced to 23.2 mg/100 g. The higher loss (10.6%) occurred when sesame seeds were roasted at 250 °C (Table 4). Because both reduction [32] and increase [33] for γ-tocopherol have been reported, it may be assumed that roasting can affect it differently depending on the temperature.

Sesamin and sesamolin are the two major lignans detected in sesame oil. They exert many health-promoting benefits, such as anti-inflammatory, antioxidant, hypocholesterolemia, neuroprotective and antihypertensive activities [34]; being important to investigate their changes after roasting process. Sesamin was the major component in the unsaponifiable of sesame oil. Variation in sesamin and sesamolin contents is also given in Table 4. Statistical analysis revealed that sesamolin was more affected (*p* < 0.001) by heat processing than sesamin (*p* < 0.01). The average lignan levels in oil before roasting were as follow: sesamin (393 mg/100 g); sesamolin (203 mg/100 g); these values were within the range already published [29,35]. Both were reduced to 344 and 147 mg/100 g, when seeds were roasted at 250 and 300 °C, respectively. The reduction rate of sesamolin (27.5%) was greater than that of sesamin (12.5%). This could be related to the observed thermal degradation of sesamolin into other phenolic compounds which have been reported during roasting [36]. This can support the observed increase TPC with increasing in roasting temperature (Table 4), describing a linear progression (*y* = 1.39 *x* + 1474; R^2^ = 0.90. The persistence of the increase in TPC at high temperature indicates that sesame phenolics are heat resistant. In our experimental conditions, treated seeds with high temperature showed a significant increase of 21.5% in TPC compared to USSO. Several authors reported improvements in TPC by heat treatment mainly after roasting [37,38].

The results for total and individual phytosterols are detailed in Table 5.

Chromatographic quantification revealed that the main sterol constituents before and after heat processing were β-sitosterol (48.4–50.6%), campesterol (31.4–34.2%), ∆-5-avenasterol (10.7–11.8%), stigmasterol (5.1–5.2%) and small amounts (<2%) for other sterols. These values are in agreement with those previously published [39]. Roasting temperature affected (*p* < 0.05) the total and all of the individual sterols. In fact, a slight decrease was observed in the individual phytosterols of roasted samples, varied between 2.7% and 11.1%. An important decrease was observed in both total phytosterols (17.4%) and ∆-7-avenasterol (36.4%). Reduction in sterols level can result from their degradation by hydrolysis, oxidation or disoperation at high temperatures [40]. ∆-5-Avenasterol was the only phytosterol that increased (9.33%) compared to the value of untreated sesame (*y* = −0.003 *x* + 10.65; R^2^ = 0.97). It has been shown that ∆-5- and ∆-7-avenasterols were increased whereas β-sitosterol and campesterol were decreased in white and brown sesame seeds roasted at 180 °C for 30 min [40].

Total sterol content of sesame was found to be high (>800 mg/100 g) compared to some other vegetable oils, such as olive, sunflower, soya and peanut [39,41]. Knowing that some sterols, including ∆-5-Avenasterol, have an anti-polymerization effect, which participates during heat treatment to the protection of oil compounds from oxidation [41], in our samples ∆-5-Avenasterol was increased in heated samples, further supporting the stability of sesame oil at high temperatures.

### 2.4. Oil Initial Quality and Antioxidant Potential of Sesame Oils

The oxidation level of oils before and after roasting was evaluated by their specific absorption, *p*-anisidine value and Rancimat method. The results are detailed in Table 6.

The *p*-AnV value is characteristic of secondary peroxidation or unsaturated aldehydes production. In USSO, its value was within the range of those already reported [16,27]. At 300 °C, it increased until 11.8 (12 times higher than that of unroasted seeds). This value was lower than that previously reported for sesame oils obtained from seeds roasted at 250 °C for 30 min in a domestic electric oven. The increase of this parameter is due to the important degradation of hydroperoxides primary products of lipid oxidation during heating leading to the release of carbonyl compounds [16]. The increase was linearly proportional to the temperature increase (*y* = 22.83*x* + 47.03, R^2^ = 0.83). The same correlation between *p*-anisidine value and the roasting was previously observed [27]. One of the most precise and reliable ways to evaluate the oxidative status of oils is by the determination of the *p*-AnV [34,42]. An ideal *p*-AnV for fresh frying oil is 4.0, with a maximum acceptable value of 6.0, according to INSO standards [43]. As a result, it seemed that roasting at temperatures up to 210 °C resulted in an oil with satisfactory quality.

The specific absorption at 232 nm (K232) and 268 nm (K268) indicates the production of conjugated dienes from PUFA [41] and conjugated trienes [44]. The increase in roasting temperature yielded a significant (*p* < 0.001) increase in both K232 and K268, showing that there was an increase in primary and secondary oxidation products. When the temperature was above 250 °C, the increase was more important. This confirms the results obtained with IV and RI previously discussed. A high and positive correlation was found between K232, K268 and the roasting temperature *y* = 0.009 *x* + 2.66, R^2^ = 0.76 and *y* = 0.002 *x* + 0.48, R^2^ = 0.73, respectively. Increases in the lipid oxidation parameters that we have used in this study (*p*-anisidine, K232 and K268) revealed a progressive increase in primary and secondary lipid oxidation. Based on the previous parameters, we can deduce that from 250 °C forward, there was an important lipid oxidation that can lead to the production of poor-quality oil.

The induction period (IP) of the Rancimat method expressed in hours (h) is commonly used to evaluate the potency of oils and fats to resist (under stress conditions such as air and heat) against accelerated oxidation. The IP was significantly affected (*p* < 0.001) by roasting temperature (Table 6), ranging from 5.5 to 10.5 h with a maximum in RSSO at 300 °C and a minimum in USSO. Effectively, many authors have reported that stability of RSSO was more pronounced than that of USSO [24,41]. This can be due to the newly formed compounds during the roasting process. Some observations were a synergistic action between sesamol, a newly formed phenolic obtained from the thermal degradation of sesamolin and γ-tocopherol using a model system in linoleic acid [45].

Comparatively to USSO, all the RSSO tested showed slightly higher (*p* < 0.01) antioxidant activity, which confirms the results found with the Rancimat test. Indeed, the antioxidant capacity of RSSO (319.1 mg GAE/100 g oil) at 300 °C was three times higher than that of USSO (139.9 mg GAE/100 g oil). Several studies were undertaken to evaluate the effects of seeds roasting on the antioxidant activity and TPC. Jannat’s findings for sesame oil were in agreement with our results, in which ferric reducing antioxidant power assay and TPC increased significantly as the roasting temperature increased [46]. Indeed, in nuts cashew, peanut and hazelnut, roasting treatment improved TPC as well as the antioxidant activities of the tested samples [47]. A strong correlation was found between the TPC and DPPH (R^2^ = 0.9773), as well as between the IP of Rancimat and TPC (R^2^ = 0.84), indicating that these compounds participate strongly in the prevention of oil oxidation. Lignans are natural antioxidants present in some oil seeds that help oil stability. Several lignans and derivatives, such as sesamin, sesamolin, sesaminol and sesamolinol in sesame oil, pinoresinol and acetoxypinoresinol in olive oil, secoisolariciresinol in flax seed oil and silymarin derivatives in milk thistle seed oil, have been shown to contribute to oil stability [16,48,49].

The potential use of the RSSOs as alternative natural antioxidants in the food industry to enhance product’s stability and extend their shelf life was finally evaluated using a meatballs model determined by the thiobarbituric acid (TBA) assay and metmyoglobin (metMb) reducing activity (Table 7).

The antioxidant capacity of RSSO was confirmed in meatballs after 10 days cold storage with a linear decrease observed in both TBA values and metMb reducing activity (Table 7). TPC and DPPH radical scavenging activities of sesame seed oils were shown to have significant relationships with these two oxidative markers of meatballs (R^2^ = 0.914, *p* = 0.01 between TBA and TPC and R^2^ = 0.912, *p* = 0.01 between TBA and DPPH assay; R^2^ = 0.970, *p* = 0.001 between metMb and TPC and R^2^ = 0.969, *p* = 0.0005 between metMb and DPPH assay). Oxidation affects the quality of meat and may lead to the formation of health-hazardous substances [50]. Because there are concerns about the safety of synthetic antioxidants [14], safe and natural antioxidants alternatives that can reduce lipid oxidation, increase their quality and maintain their nutritional value are actively searched for. Several plant components or extracts, including sesame lignans, have been reported to act as effective natural antioxidants in meat samples [14,15]. In this study, we showed for the first time that RSSO, particularly heat-released phenolics, might be a potentially beneficial natural additive to fresh meat products for extending shelf life during cold storage.

## 3. Materials and Methods

### 3.1. Chemical Reagents and Standards

The reagents used in this study (analytical or higher grade) were from Sigma–Aldrich (St. Louis, MO, USA) and Merck (Darmstadt, Germany): ether petroleum, hexane, ether, acetone, isooctane and ethyl acetate. Tetrahydrofuran THF, heptane, n-hexane and diethyl ether were HPLC grade from the same suppliers. Silica cartridges for SPE were Sep-Pak columns supplied by Waters (Supelco, Bellefonte, PA, USA). The internal standard used for polar compounds quantification (monostearin) was obtained from Supelco (Bellefonte, PA, USA). Free stable DPPH radical, gallic acid and sterols standards were purchased from Sigma–Aldrich (St. Louis, MO, USA). Certified fatty acids methyl ester mixture (Supelco 37 Component FAME Mix, Supelco, Bellefonte, PA, USA) was from Supelco (Trace CERT, Bellefonte, PA, USA) as was γ-Tocopherol. The internal standard [2-Methyl-2-(4,8,12-trimethyltridecyl)-chroman-6-ol(tocol)] was purchased from Matreya Inc. (State College, PA, USA).

### 3.2. Seed Roasting and Oil Extraction

White sesame seeds were purchased from a local market (Bejaia city, Algeria). Impurities such as dust, sands, stones, spoiled seeds and other extra materials were separated by sieves. The seeds were roasted at different temperatures (120, 150, 180, 210, 250 and 300 °C) for 20 min using an electric oven (Samsung: NQ50T9939BD, Manchester, UK) and then ground (GM 200; Retsch GmbH, Éragny, Luxembourg) into a fine powder. The obtained powders were extracted with petroleum ether (boiling range 40–60 °C) in a Soxhlet extractor for 6 h (AOAC, 1998). The extracts were taken to dryness to eliminate solvent in a rotary vacuum Model Bibby Scientific (Stone, UK) at 40 °C then the residual solvent was removed using a gentle nitrogen stream. Efficiency of pressing in the given temperature was expressed as the weight of collected oil on the mass basis used for extraction. Extracted oils were stored in brown glass under refrigeration until use. 

### 3.3. Physical and Chemical Properties of Extracted Oils

The moistures, densities or specific gravities, refractive indexes, saponification values and iodine values of the extracted oils were determined using AOAC methods [51]. Color parameters: lightness (L*), redness (a*) and yellowness (b*) were determined with Konica Minolta (Chroma Meter CR-400, Tokyo, Japan), calibrated in a measuring cup 7 mm high.

In detail:

Moisture was determined from 2 g at 105 °C until the weight remained constant and was calculated according to following equation.

$$\%\;\mathrm{moisture}=\frac{Wi-Wf}{Wi}\times 100$$

where: *W_i_*, initial weight of sample (2 g); and *W_f_*, final weight of sample.

To determine density, a pycnometer was weighed empty (m_0_), then weighed with 0.5 mL of distilled water (m_1_) and finally weighed with 0.5 mL of oil sample (m_2_). Pycnometers containing the sample and distilled water were placed in a water bath at a temperature of 20 °C for 20 min (AOAC, 1998). The density was determined using the following equation:

$$d=\frac{m_{2}-m_{0}}{m_{1}-m_{0}}\times 100$$

m0: mass in grams of the empty pycnometer, m1: mass in grams of the pycnometer filled with distilled water and m2: mass in grams of the pycnometer containing the oil.

The refractive index “rI” of a substance is the ratio between the speed of light (of determined wavelength) in air and the speed of this same light in this substance. 10 g of oil were first heated in a water bath (40 °C). A few drops were deposited between the prisms of the refractometer so as to completely fill the space between these prisms. After a few minutes, allowing the fat to reach thermal equilibrium, the value given by the refractometer was noted.

The saponification index is the amount of potassium hydroxide (in milligrams) required to saponify 1 g of fat. This value is all the higher since the fatty acids are of low molecular weight. Its principle remains on a reflux boiling of a test portion with a potassium hydroxide solution, followed by a titration of the excess potassium hydroxide with a hydrochloric acid solution. 2 g of oil were dissolved in 2 mL of the potassium hydroxide solution. After heating the solution under reflux for one hour, 0.5 to 1 mL of the phenolphthalein solution were immediately added to the mixture and then titrated with a hydrochloric acid solution (0.5 N) until the color pink [51] completely disappeared. The saponification value was calculated by the formula established below:

$$\mathrm{SV}=\frac{N\times 56.1\times \left(\mathrm{Vo}-V\right)}{m}$$

Vo: volume in ml of HCl used for the blank test, V: volume in ml of HCl used for the sample, m: test sample in grams and N: normality of hydrochloric acid HCl 0.5 N.

The iodine value allows the measurement of unsaturation degree of fat by determining the amount of iodine in grams which fixes it on the double bonds present in 100 g of lipids. It is determined using the Wijs reagent and with titration with a sodium thiosulfate solution in the presence of starch as a color indicator. In an Erlenmeyer flask, 0.3 g of the oil was mixed with 25 mL of Wijs reagent. 20 mL of potassium iodide (10%) and 150 mL of distilled water were added to this mixture and the whole was well-shaken and then incubated for 1 h in the dark. Titration with the 0.1 N sodium thiosulfate solution (Na_2_S_2_O_3_) was carried out in the presence of a few drops of starch paste as an indicator until the black-blue color disappeared. The iodine value was given by the following formula:

$$I(g/100g)=\frac{{(V}_{1}-V_{0})}{W}\times 1.29$$

with N: Normality of the solution (0.1 N), V_0_: Volume of Na_2_S_2_O_3_ (mL) necessary to titrate the blank test, V_1_: Volume of Na_2_S_2_O_3_ (mL) necessary to titrate the sample and W: Test sample (g).

The color parameters were determined using a Konica Minolta type colorimeter (Chroma Meter CR-400, Tokyo, Japan). The color parameters L*, a* and b* represent the clarity, green-red color and blue-yellow color, respectively.

L*: represents the clarity where L* = 0 (black), L* = 100 (white).
a*: represents the green-red color, from green (−a*) to red (+a*).
b*: represents the blue-yellow color, from blue (−b*) to yellow (+b*).

The effect of seed roasting on the color of oils was achieved with a Konica Minolta device, calibrated on a white background. A plastic cup with a diameter of 3 cm was filled with the oil to be analyzed, to take the readings. The color measurement was carried out in the dark and the values of the different color parameters (*L*, *a*, *b*) were given directly by the colorimeter at the same time.

### 3.4. Oil Initial Quality

Specific extinctions at 232 and 268 wavelengths were determined according to the CEE N.2568/91 regulation [52]. Anisidine value was determined following the ISO 6885.2 method. The oxidative stability of oils was measured according to the ISO 6886 method [53] using a Rancimat 982 apparatus (Metrohm CH, Zofingen, Switzerland).

### 3.5. Determination of Triglyceride Derivatives

Estimation of tryglycerides derivatives was carried out using high-performance size-exclusion chromatography (HPSEC) described previously [31] with refractive index detection (IR) (31), using monostearin as an internal standard. The polar triglyceride fraction was separated by solid phase extraction (silica, 1 g) and separated on a Phenomenex (Torrance, CA, USA) column (Phenogel, 100 Å, 5 µm, 7.8 × 600 mm), using tetrahydrofuran as an eluent (1 mL/min).

### 3.6. Fatty Acids (FA) Composition

The FA composition of roasted and unroasted sesame oil was determined after alkaline trans-esterification by gas-liquid chromatography with flame ionization detection (GC-FID) as detailed in Regulation EEC 2568/91 [52]. Fatty acid methyl esters (FAME) and standards were analyzed using a Chrompack CP 9001 gas chromatograph (Chrompack, Middelburg, The Netherlands). Identification of the FAME was based on the comparison of their retention times with those of the FAME standard mixture. The quantification was made by using peak areas in electronic units and the fatty acid content was expressed in relative percentage of each fatty acid.

### 3.7. Tocopherols and Lignans Contents

Tocopherols and lignans (sesamin and sesamolin) content determinations were carried out according to the method developed previously [54]. The compounds were identified by chromatographic comparisons with authentic standards for the tocotrienol, or with fractionated compounds from oil (sesamin and sesamolin), and by their UV spectra comparison, using a PDA detector (Jasco MD-2015 Plus, Tokyo, Japan). Quantification was based on the fluorescence signal response, using the internal standard method, with the standards calibrated on the basis of their chromatographic purity and published extinction coefficients [55,56].

### 3.8. Sterol Content and Profile

The sterol profile of both unroasted and roasted sesame oils was determined according to the European Union Regulation 2568:1991 using GC-FID [17]. The concentration of each individual sterol, in mg/100 g of oil, was calculated on the basis of the internal standard.

### 3.9. Total Phenolic Content

Total phenolic content (TPC) was determined with the Folin–Ciocalteu method. The oil was dissolved with ethyl acetate and incubated 5 min at room temperature in the dark with the Folin–Ciocalteu reagent. After addition of Na_2_CO_3_ and dilution with deionized water, the mixture was left for 90 min at room temperature in the dark, centrifuged and the absorbance was read at 765 nm. Calibration curves were performed with gallic acid and the results of total phenolics were expressed in mg gallic acid equivalent/100 g of oil.

### 3.10. Free Radical Scavenging Activity

The antioxidant activity of the roasted and unroasted oils was evaluated by assessing their ability to scavenge the 2,2′-diphenyl-1-picrylhydrazyl radical. To 500 µL of sample solution in ethyl acetate were added 3.5 mL of DPPH agent (2.5 mg diluted in 100 mL ethyl acetate). The mixture was homogenized, incubated 30 min at room temperature in the dark, centrifuged during 5 min at 5000 rpm and then the absorbance was read at 517 nm. Calibration curves were performed with gallic acid, and the antioxidant activity was expressed in mg gallic acid equivalent/100 g of oil.

### 3.11. Evaluation of the Oxidative Stability of Meatballs

The meat samples were prepared as described by [15] with the addition of 50 g of (un)roasted sesame oil and kept aerobically in the refrigerator at 3 ± 1 °C throughout the research period of 10 days. The thiobarbituric acid (TBA) assay was performed using a spectrophotometer (BioTek ELX800 Absorbance MicroplateReader, BioTek Instruments, Colmar, France) at 532 nm as described previously [16]. The evaluation of the metmyoglobin (metMb) reducing activity was performed using a spectrophotometer (BioTek ELX800 Absorbance MicroplateReader, BioTek Instruments, Colmar, France) at 580 nm [14].

### 3.12. Statistical Analysis

All experiments were carried out in triplicate and the values are expressed in mean ± standard deviation. Results were analyzed for variance (ONE-WAY ANOVA), followed by multiple comparisons of means (FISHER LSD test) using the software Statistica version 7.1 (StatSoft, Inc., Tulsa, OK, USA). The differences are significant at *p* < 0.05.

## 4. Conclusions

The effect of the roasting process on white sesame seeds was evaluated in this study. As the roasting temperature increased, the composition and content of fatty acids and antioxidant phytochemicals changed significantly. Based on our data, roasting improved the oxidative stability, the TPC and the antiradical activity of the extracted oil. However, it decreased some the phytochemicals such as lignans, tocopherol and sterols. A slight increase in TFAs, as well as triglyceride and diglycerides dimmers, was recorded in seeds oil roasted at high temperatures. The results revealed that the optimal roasting temperature for obtaining high nutritional grade oil within the permissible values was 210 °C. The strong antioxidative efficiency of roasted seeds oil may lead to increased nutritional quality from the use of this roasted sesame oil seed in plant-based diets. The unsaponifiable components (including lignans and sterols) extracted from roasted seeds in optimum conditions can be used as beneficial natural additives to fresh meat products to extend shelf life during cold storage. The results of this study may help to increase the nutritional value of plant-based diets by facilitating the use of roasted sesame seed oil and its components.

## Figures and Tables

**Table 1:** Physical and chemical parameters of unroasted and roasted sesame oils.

	Samples						
Parameters	USSO	RSSO 150	RSSO 180	RSSO 210	RSSO 250	RSSO 300	*p*
Oil moisture (%)	2.55 ± 0.03 e	1.58 ± 0.03 d	1.15 ± 0.03 c	0.95 ± 0.03 c	0.61 ± 0.03 b	0.15 ± 0.03 a	<0.001
Efficiency of pressing (%)	33.5 ± 0.43 a	39.1 ± 1.89 b	41.6 ± 0.9 c	43.9 ± 1.29 d	62.5 ± 1.31 f	56.3 ± 2.01 e	<0.001
Specific gravity g/cm^3^	0.919 ± 0.01 a	0.920 ± 0.04 a	0.921 ± 0.03 b	0.922 ± 0.02 b	0.921 ± 0.03 b	0.923 ± 0.01 c	0.058
Refractive index	1.476 ± 0.02 b	1.476 ± 0.001 b	1.475 ± 0.03 b	1.473 ± 0.01 ab	1.468 ± 0.02 a	1.461 ± 0.03 a	<0.05
Iodine value (gI_2_/100 g oil)	113.11 ± 0.43 e	113.91 ± 0.84 f	112.4 ± 0.96 d	110.81 ± 0.73 c	105.41 ± 0.52 b	103.17 ± 0.73 a	<0.01
Saponification value (g KOH/100 g oil)	185.45 ± 0.78 a	186.12 ± 0.15 b	189.87 ± 0.54 c	192.15 ± 0.42 d	195.67 ± 1.34 e	200.05 ± 1.08 f	<0.01
Parameter L*	48.66 ± 0.01 f	44.52 ± 0.1 c	47 ± 0.02 d	47.24 ± 0.03 e	42.87 ± 0.05 a	43.35 ± 0.01 b	<0.01
a*	−3.97 ± 0.01 a	−1.86 ± 0.04 d	−2.22 ± 0.01 c	−2.37 ± 0.00 b	0.24 ± 0.01 e	1.80 ± 0.01 f	<0.001
b*	13.33 ± 0.01 a	17.29 ± 0.01 c	17.94 ± 0.01 d	18.2 ± 0.01 e	16.49 ± 0.01 b	17.32 ± 0.01 c	<0.001

Data are expressed as means ± standard deviations (*n* = 3) of the measurements. Different letters (a < b < c < d < e < f) in the same row show statistical differences between them (*p* < 0.05). The effects of roasting on the parameters are given by the *p*-value (*p*).

**Table 2:** Fatty acids composition expressed in % (g/100 g of oil) of unroasted and roasted sesame oils at different temperatures.

Fatty Acid	Samples						
	USSO	RSSO 150	RSSO 180	RSSO 210	RSSO 250	RSSO 300	*p*
C14:0	0.02 ± 0.00 a	0.02 ± 0.00 a	0.02 ± 0.00 a	0.02 ± 0.00 a	0.02 ± 0.00 a	0.02 ± 0.00 a	0.352
C16:0	9.21 ± 0.08 a	9.28 ± 0.04 b	9.22 ± 0.01 ab	9.3 ± 0.00 c	9.37 ± 0.03 d	9.38 ± 0.02 d	0.112
C17:0	0.07 ± 0.00 a	0.06 ± 0.00 a	0.06 ± 0.00 a	0.06 ± 0.00 a	0.06 ± 0.00 a	0.06 ± 0.00 a	0.132
C17:1	0.02 ± 0.00 a	0.02 ± 0.00 a	0.02 ± 0.00 a	0.02 ± 0.00 a	0.02 ± 0.00 a	0.02 ± 0.00 a	0.105
C18:0	6.06 ± 0.04 a	6.17 ± 0.00 b	6.16 ± 0.00 b	6.14 ± 0.01 b	6.14 ± 0.02 b	6.15 ± 0.03 b	0.354
C18:1_t9	0.01 ± 0.00 a	0.08 ± 0.00 b	0.08 ± 0.00 b	0.16 ± 0.00 c	0.22 ± 0.00 d	0.29 ± 0.00 e	0.036
C18:1_c9	37.55 ± 0.17 c	37.16 ± 0.01 b	37.05 ± 0.02 ab	37.13 ± 0.02 ab	37.07 ± 0.08 ab	37.00 ± 0.03 a	0.765
C18:1_c11	0.93 ± 0.01 b	0.69 ± 0.00 a	0.91 ± 0.01 b	0.92 ± 0.00 b	0.91 ± 0.00 b	0.92 ± 0.00 b	0.376
C18:2_t9t12	0.00 ± 0.00 a	0.00 ± 0.00 a	0.00 ± 0.00 a	0.00 ± 0.00 a	0.01 ± 0.00 b	0.02 ± 0.00 c	0.055
C18:2_c9t12	0.05 ± 0.00 a	0.09 ± 0.00 b	0.09 ± 0.00 b	0.16 ± 0.00 c	0.22 ± 0.00 d	0.29 ± 0.00 e	0.047
C18:2_t9c12	0.00 ± 0.00 a	0.03 ± 0.00 b	0.04 ± 0.01 b	0.11 ± 0.01 c	0.17 ± 0.00 d	0.24 ± 0.01 e	0.028
C18:2_c9c12	44.12 ± 0.2 c	44.35 ± 0.03 cd	44.39 ± 0.01 d	44.13 ± 0.03 c	43.84 ± 0.07 b	43.62 ± 0.03 a	0.115
C18: 3_N3	0.8 ± 0.00 d	0.38 ± 0.00 c	0.38 ± 0.00 c	0.37 ± 0.00 b	0.36 ± 0.01 a	0.36 ± 0.00 a	0.056
C20:0	0.66 ± 0.01 a	0.66 ± 0.01 a	0.68 ± 0.03 b	0.66 ± 0.00 a	0.66 ± 0.00 a	0.66 ± 0.00 a	0.126
C20:1c9	0.18 ± 0.00 a	0.17 ± 0.00 a	0.18 ± 0.00 a	0.17 ± 0.00 a	0.17 ± 0.00 b	0.17 ± 0.00 ab	0.345
C21:0	0.01 ± 0.00 a	0.01 ± 0.00 a	0.01 ± 0.00 a	nd	nd	0.01 ± 0.00 a	0.104
C22:0	0.13 ± 0.00 a	0.14 ± 0.00 a	0.14 ± 0.00 a	0.14 ± 0.00 a	0.14 ± 0.00 a	0.14 ± 0.00 a	0.543
C24:0	0.09 ± 0.00 c	0.06 ± 0.00 a	0.09 ± 0.00 c	0.09 ± 0.00 c	0.08 ± 0.00 b	0.09 ± 0.00 c	0.128
C24:1	0.1 ± 0.00 b	0.03 ± 0.00 a	0.02 ± 0.00 a	0.04 ± 0.00 a	0.04 ± 0.00 a	0.04 ± 0.00 a	0.132
SFA	16.24 ± 0.12 a	16.40 ± 0.03 ab	16.37 ± 0.04 a	16.41 ± 0.04 ab	16.47 ± 0.03 c	16.50 ± 0.03 c	0.084
MUFA	38.94 ± 0.14 b	38.25 ± 0.45 a	38.35 ± 0.18 a	38.45 ± 0.02 a	38.38 ± 0.05 a	38.32 ± 0.03 a	0.065
PUFA	44.52 ± 0.2 c	44.75 ± 0.03 d	44.79 ± 0.2 e	44.52 ± 0.03 c	44.22 ± 0.06 b	44.00 ± 0.03 a	0.076
Trans fatty acid	0.05 ± 0.00 a	0.21 ± 0.01 b	0.28 ± 0.01 c	0.43 ± 0.01 d	0.62 ± 0.04 e	0.84 ± 0.01 f	<0.05

“t” and “c” mean cis and trans, respectively; nd: not detected Mean ± SD, *n* = 3 expressed in relative percentage of total fatty acids. Different letters (a < b < c < d < e < f) in the same row show statistical differences between them (*p* < 0.05). The effects of roasting on the parameters are given by the *p*-value (*p*). SFA, MUFA and PUFA, respectively, denoted saturated, monounsaturated and polyunsaturated fatty acids.

**Table 3:** Distribution of triglyceride derivatives expressed in relative % and total amount (g/100 g oil) in oils.

	Samples						
Compounds	USSO	RSSO 150	RSSO 180	RSSO 210	RSSO 250	RSSO 300	*p*
TGP + TGD	0.1 ± 0.00 a	6.7 ± 0.9 f	4.7 ± 0.64 b	5.2 ± 097 c	5.4 ± 0.54 d	9.5 ± 0.01 e	0.045
oxTGM	18.8 ± 0.67 a	22.5 ± 0.03 b	25.6 ± 0.02 e	22.1 ± 0.06 b	23.7 ± 0.13 d	22.9 ± 0.21 c	0.043
DG	37.4 ± 0.01 b	34.7 ± 0.001 a	34.2 ± 0.46 a	38.3 ± 0.51 c	38.4 ± 0.72 c	38.2 ± 0.83 c	0.053
FFA	38.1 ± 0.71 d	27 ± 0.84 b	27.4 ± 0.16 b	29.1 ± 2.03 c	27.1 ± 1.52 b	24.3 ± 0.13 a	<0.05
Total triglyceride derivatives (g/100 g oil)	5.7 ± 0.4 a	6.4 ± 0.5 bc	6.3 ± 0.3 b	6.3 ± 0.8 b	6.5 ± 0.1 c	6.7 ± 0.01 d	<0.05

Data are expressed as means ± standard deviations (*n* = 3) of the measurements. Different letters (a < b < c < d < e < f) in the same row show statistical differences between them (*p* < 0.05). The effects of roasting on the parameters are given by the *p*-value (*p*). Abbreviations: TGP, triglyceride polymers; TGD, triglyceride dimers; oxTGM, oxidized triglyceride monomers; DG, diglycerides; and FFA, free fatty acids.

**Table 4:** γ-tocopherol, lignan (sesamin, sesamolin) and total phenolic compounds (TPC) contents in row and roasted sesame oils at different temperatures.

	Samples						
	USSO	RSSO 150	RSSO 180	RSSO 210	RSSO 250	RSSO 300	*p*
*γ*-tocopherol (mg/100 g)	25.93 ± 1.5 b	24.89 ± 1.5 ab	24.37 ± 0.2 ab	24.6. ± 1.5 ab	23.18 ± 1.6 a	24.87 ± 0.7 ab	<0.05
sesamolin (mg/100 g)	202.92 ± 9.5 f	201.351 ± 14.3 e	190.11 ± 14.8 d	182.30 ± 9.5 c	163.37 ± 9.9 b	147.12 ± 2.68 a	<0.001
sesamin (mg/100 g)	393.25 ± 14.7 e	392.4 ± 4 d	364.89 ± 10.8 c	361.56 ± 9.3 c	344.19 ± 13.73 b	354.8 ± 6.99 a	<0.01
TPC	152.2 ± 1.73 a	160.78 ± 1.8 b	169.6 ± 1.5 c	177.5 ± 1.8 d	183.33 ± 1.53 e	193.87 ± 1.1 f	<0.01

Data are expressed as means ± standard deviations (*n* = 3) of the measurements. Different letters (a < b < c < d < e < f) in the same row show statistical differences between them (*p* < 0.05). The effects of roasting on the parameters are given by the *p*-value (*p*). TPC: Total phenolic compounds expressed in mg gallic acid equivalent per 100 g of oil.

**Table 5:** Sterol contents (relative% and total amount) of raw and roasted sesame seed oils at different temperatures.

	Samples						
	USSO	RSSO 150	RSSO 180	RSSO 210	RSSO 250	RSSO 300	*p*
Campesterol	33.39 ± 0.14 c	34.06 ± 0.14 e	34.24 ± 0.04 f	33.8 ± 0.04 d	32.64 ± 0.03 b	31.38 ± 0.05 a	0.043
Stigmasterol	5.17 ± 0.03 b c	5.11 ± 0.06 b	5.06 ± 0.01 a	5.1 ± 0.03 ab	5.2 ± 0.02 c	5.2 ± 0.02 c	0.039
β-Sitosterol	49.49 ± 0.05 b	48.44 ± 0.15 a	48.42 ± 0.09 a	48.65 ± 0.05 ab	49.47 ± 0.02 b	50.55 ± 0.01 c	<0.05
∆-5-Avenasterol	10.68 ± 0.03 a	11.22 ± 0.07 b	11.22 ± 0.1 b	11.33 ± 0.06 c	11.54 ± 0.05 d	11.78 ± 0.1 e	0.073
∆-7-Stigmasterol	0.99 ± 0.04 d	0.95 ± 0.07 c	0.88 ± 0.02 a	0.88 ± 0.04 a	0.93 ± 0.03 b	0.88 ± 0.00 a	<0.05
∆-7-Avenasterol	0.22 ± 0.03 d	0.19 ± 0.04 b	0.14 ± 0.02 a	0.2 ± 0.04 c	0.2 ± 0.03 c	0.19 ± 0.03 b	<0.05
Total sterols (mg/100 g)	862.9 ± 8.9 e	896.4 ± 10.9 f	824.5 ± 3.2 d	819.1 ± 9.69 c	785.5 ± 5.21 b	740.2 ± 8.54 a	<0.05

Data are expressed as means ± standard deviations (*n* = 3) of the measurements. Different letters (a < b < c < d < e < f) in the same row show statistical differences between them (*p* < 0.05). The effects of roasting on the parameters are given by the *p*-value (*p*).

**Table 6:** Evaluation of the oxidative stability and antioxidant activity of raw and roasted sesame seed oils at different temperatures.

	Samples						
Parameters	USSO	RSSO 150	RSSO 180	RSSO 210	RSSO 250	RSSO 300	*p*
*p*-anisidine value (*p*-AnV)	0.94 ± 0.12 a	2.76 ± 0.17 b	4.4 ± 0.28 c	5.81 ± 0.37 d	9.63 ± 0.86 e	11.81 ± 0.75 f	<0.0001
K 232	3.12 ± 0.04 a	3.87 ± 0.22 c	3.53 ± 0.29 b	4.28 ± 0.25 d	5.3 ± 0.29 e	6.00 ± 0.26 f	<0.001
K 268	0.65 ± 0.02 a	0.77 ± 0.05 b	0.78 ± 0.06 b	0.92 ± 0.06 c	1.27 ± 0.17 d	1.49 ± 0.10 e	<0.001
Rancimat (h)	5.51 ± 0.19 a	5.61 ± 0.27 a	5.94 ± 0.08 b	6.11 ± 0.17 c	7.11 ± 0.14 d	10.5 ± 0.2 e	<0.05
DPPH	139.86 ± 4.58 a	160.78 ± 1.8 b	169.6 ± 1.5 c	177.5 ± 1.8 d	183.33 ± 1.53 e	193.87 ± 1.1 f	<0.01

Data are expressed as means ± standard deviations (*n* = 3) of the measurements. Different letters (a < b < c < d < e < f) in the same row show statistical differences between them (*p* < 0.05). The effect of roasting on the parameters are given by the *p*-value (*p*). The specific absorption at 232 nm (K232) and 268 nm (K268) indicates the production of conjugated dienes from PUFA. DPPH free radical scavenging antioxidant activity is expressed in mg gallic acid equivalent/100 g of oil.

**Table 7:** Effects of addition of raw and roasted sesame seed oils at different temperatures on TBA (thiobarbituric acid) and metmyoglobin (metMb) reducing activity value in meatballs after 10 days cold storage.

Parameters	Samples						
	USSO	RSSO 150	RSSO 180	RSSO 210	RSSO 250	RSSO 300	*p*
TBA	0.53 ± 0.08 a	0.50 ± 0.06 a	0.47 ± 0.04 a	0.41 ± 0.06 ab	0.33 ± 0.04 bc	0.23 ± 0.04 c	<0.05
metMb	59.37 ± 4.98 a	49.90 ± 2.26 b	45.22 ± 3.02 bc	44.63 ± 1.55 b	40.27 ± 1.10 c	35.33 ± 2.52 d	<0.05

TBA expressed as mg malonaldehydes/kg of meatball samples. metMbis expressed in %. Results are mean ± SD, *n* = 3. Values followed by the same letters within each row are not significantly different (*p* > 0.05). The effects of roasting on the parameters are given by the *p*-value (*p*).

## Data Availability

All the data supporting the findings of this study are included in this article.
